# Universal Utilization of Optimally Timed First Obstetric Ultrasound Among Pregnant Women in a Low‐Middle Income Country: A Multicenter Prospective Study

**DOI:** 10.1155/ogi/5797924

**Published:** 2025-12-23

**Authors:** Sisay Kirba, Abdulfetah Abdulkedir Abdosh, Ekram Muhammedasrar, Abraham Fessehaye Sium

**Affiliations:** ^1^ Department of Obstetrics and Gynecology, St. Paul’s Hospital Millennium Medical College, Addis Ababa, Ethiopia, sphmmc.edu.et

**Keywords:** early ultrasound, LMICs, obstetric ultrasound, obstetrics, prenatal care

## Abstract

**Background:**

Currently, the World Health Organization guidelines recommend universal utilization of optimally timed first obstetric ultrasound (defined as ultrasound before 24 weeks) in low‐middle income countries. However, there is inadequate evidence on this topic from these countries. Our study aimed to determine the rate of utilization of optimally timed first obstetric ultrasound among pregnant women who gave birth at three public hospitals in Ethiopia.

**Methods:**

This was a multicenter prospective cross‐sectional study conducted on the utilization of optimally timed first obstetric ultrasound among pregnant women who delivered at public hospitals in Ethiopia in 2020. Data were collected prospectively using a structured questionnaire. Data were analyzed using SPSS Version 20. Simple descriptive statistics, chi‐squared test, and multiple regression analysis were performed as appropriate. *p* value less than 0.05 and adjusted odds ratio (AOR) with 95% CI were used to present result significance.

**Results:**

A total of 385 participants were included in this study. Approximately 67.5% of pregnant mothers had optimally timed 1^st^ ultrasound. Compared to those having antenatal care (ANC) at health centers, those who started their ANC at private clinic were 1.7 times (95 CI: 1.8–7.9) more likely to have optimally timed ANC. Those who had their 1^st^ ANC at private hospital were 3.2 times (95 CI: 1.8–7.9) more likely to have optimally timed 1^st^ prenatal care. Those who had their first ANC at private health institutions and government hospitals were much more likely to have optimally timed 1^st^ prenatal ultrasound with the following AOR for government hospital and private MCH centers: AOR = 7.4 (95 CI: 2.7–23) and AOR = 4.9 (95 CI: 2.8–14.1), respectively. Those who had previous major obstetric problem were 5.2 times (95 CI: 2.7–9.9) more likely to have optimally timed ultrasound than those without major previous obstetric problem.

**Conclusion:**

We found that one‐third of pregnant women did not utilize optimally timed first obstetric ultrasound, despite obstetric ultrasound services being accessible at public health institutions. Place of first ANC contact and presence of prior major obstetric complication were associated with utilization of optimally dated first obstetric ultrasound.

## 1. Introduction

Antenatal care (ANC) is a basic health care that is provided to pregnant mothers during pregnancy. ANC is a time proven important tool that decreases fetomaternal morbidity and mortality [1]. It has three core scopes: early detection of risks that increase morbidity and mortality both in the fetus and the mother, preventing and managing pregnancy‐related complications with treatments and immunizations, and promoting health through nutrition guidance, lifestyle modification, and education on birth preparedness and danger signs [2]. In the first scope, elevated risk is identified by basic history and physical examination and by doing some basic and some specialized investigation [3]. This is very important objective of ANC, and it is preferably to be done as early in the pregnancy as possible so that the pregnant mother can get specialized care that minimizes the increased morbidity and mortality in the fetomaternal condition. The other reason it has to be done as early as possible is that in the event of fetal conditions that have no acceptable and available therapy and or fetal conditions that are lethal, pregnancy termination can be provided safely [4]. This is also important in the event of maternal conditions which are worsened by pregnancy and endangering the life of the mother [5]. Evaluations and workups done early in pregnancy are classified as basic and specialized [6].

The woman that needs specialized care is identified by appropriate history and physical examination and baseline workup [7]. Among the basic evaluations that should be carried out early during the course of the pregnancy is obstetric ultrasound (US). Obstetric US early in the pregnancy has basic objective of dating the pregnancy, localizing the pregnancy, and assessing the viability of the pregnancy. Another simple noninvasive diagnostic evaluation during ANC is doing fetal anatomic scanning. Fetal anatomic scan can diagnose most of the major congenital anomalies when done at the appropriate GA (18–20 weeks) [8, 9]. In its latest recommendation, WHO recommends all pregnant woman should have the first obstetric US before 24 weeks [5].

In Ethiopia, the first ANC visit is usually done at health centers, and the risk identification evaluation and workup are done by obstetric healthcare providers at health centers who are almost always midwives, health officers, or nurses [7, 10–12].

In the current practice in Addis Ababa (the capital city of Ethiopia), early obstetric US is mostly done by obstetricians and radiologists. Although multiple studies have documented the timing of first ANC follow‐up and the quality of ANC follow‐up [13–17], there is no prior study that looked into the utilization of optimally timed first obstetric US among Ethiopian pregnant women. The objective of this study was to determine the mean timing of first obstetric US among pregnant women who attended ANC at the abovementioned public hospitals and factors associated with late timing of first obstetric US (first US beyond 24 weeks of gestation).

## 2. Methods

### 2.1. Study Design, Setting, and Period

This was a cross‐sectional study conducted on the timing of first obstetric US among women who gave birth at three public hospitals (St. Paul’s Hospital Millennium Medical College [SPHMMC], Ras Desta Damtu Hospital, and Menellik‐II hospital) in Addis Ababa, Ethiopia. The study was conducted over a period of 3 months (October to December 2020). SPHMMC is a leading medical college in Ethiopia that provides various specialty and subspecialty care and training in Ethiopia. The Department of Obstetrics and Gynecology at this hospital runs the biggest maternal–fetal medicine unit in the country providing advanced care for high‐risk pregnant women including screening for congenital anomalies, genetic counseling and testing, and fetal diagnostic and therapeutic interventions including intrauterine transfusion. The other two hospitals (study sites) are affiliates of St. Paul’s Hospital, and the majority of the obstetric service deliveries at these hospitals are carried out by SPHMMC Ob‐Gyn residents and faculty.

We included both high‐risk and low‐risk pregnant women who had their deliveries attended at the three public hospitals and volunteered to participate in the study. We excluded those with incomplete data and missing key variables (such as timing of first US, undocumented status of presence of prior serious medical conditions, and undocumented place of first ANC contact).

### 2.2. Sample Size and Sampling Procedure

Using single proportion formula by assuming a 50% prevalence of optimally timed first obstetric US, 5% error of margin, and 95% confidence interval, the calculated sample size was *N* = 385. No systematic sampling technique was employed.

### 2.3. Data Collection

Data were collected using a structured questionnaire prepared in English. The questionnaire was pretested before data collection and modified accordingly. We collected sociodemographic data and obstetric characteristics including timing of first obstetric US.

### 2.4. Data Analysis

Data were analyzed using SPSS 20 statistical software. Simple descriptive statistics with mean and SD were used for numerical variables. Bivariate regression analysis was carried out, and variables with *p* value less than 0.2 (considered potential confounders) were entered into multivariate regression analysis; factors associated with late timing (which is not optimal timing) of first obstetric US were further analyzed. *p* value less than 0.05 and adjusted odds ratio (AOR) with 95% CI were used to present result significance.

### 2.5. Ethical Consideration

This research was approved by the Ethical Review Committee of SPHMMC with Ref. No. PM23/232. Written informed consent was obtained from study participants. Confidentiality and data anonymity were maintained throughout the data collection.

## 3. Results

A total of 385 women were included in the final analysis. The mean gestational age for the 1^st^ ANC was 3.1 months with SD of 1.3. Approximately 67.5% of pregnant mothers had optimally timed 1^st^ US (32.5% of pregnant mothers did not have optimally timed 1^st^ prenatal US).

The mean age of respondents was 28.3 years with the youngest age being 18 years and the oldest being 40 years (Table 1). From the study participants, 76.1% of them were from Addis Ababa and 23.9% were from outside Addis Ababa. When it comes to educational status, 14.3% of respondents did not go to school while 41% were unemployed. Approximately 40.3% of the pregnant mothers were primigravida. The majority (48.1%) of respondents started their ANC at health centers (Table 2).

**Table 1:** Sociodemographic characteristics of pregnant women who had their deliveries attended in public hospitals in Ethiopia, 2020, *N* = 385.

Variable		Number	Percent
1. Address	Addis Ababa	293	76.1
	Outside Addis Ababa	92	23.9

2. Marital status	Single	43	11.2
	Married	342	88.8

3. Religion	Orthodox Christian	155	40.3
	Protestant Christian	107	27.8
	Muslim	116	30.1
	Other	7	1.8

4. Level of education	Uneducated	55	14.3
	Completed primary school	66	17.1
	Completed secondary school	107	27.8
	College diploma	91	23.6
	College degree	57	14.8
	Master’s degree	9	2.3
	Doctorate degree	0	0

5. Employment status	Unemployed	158	41
	Government employee	153	39.7
	Private employee	59	15.3
	Run self‐business	15	3.9

6. Total monthly income in birr	< 5000	95	24.7
	5000–10,000	103	26.8
	10–20,000	161	41.8
	20–50,000	24	6.2
	> 50,000	2	0.5

**Table 2:** Obstetric characteristics of pregnant women who had their deliveries attended at three public hospitals in Ethiopia, 2020, *N* = 385.

Variable		Number	Percent
1. Parity	Primigravid	155	40.3
	Multigravid	230	59.7

2. Known health problems before this pregnancy	Yes	187	48.5
	No	198	51.5

3. Health problems during this pregnancy	Yes	220	57.1
	No	165	42.9

4. Previous history of major obstetric problem during pregnancy	Yes	130	33.5
	No	100	26.5

5. Planned pregnancy	Yes	311	80.8
	No	74	19.2

6. Place of 1^st^ ANC visit	Health center	185	48.1
	Private clinic	41	10.6
	Private hospital	57	14.8
	Government hospital	39	10.1
	Private MCH center	63	16.4

**Figure 1:**
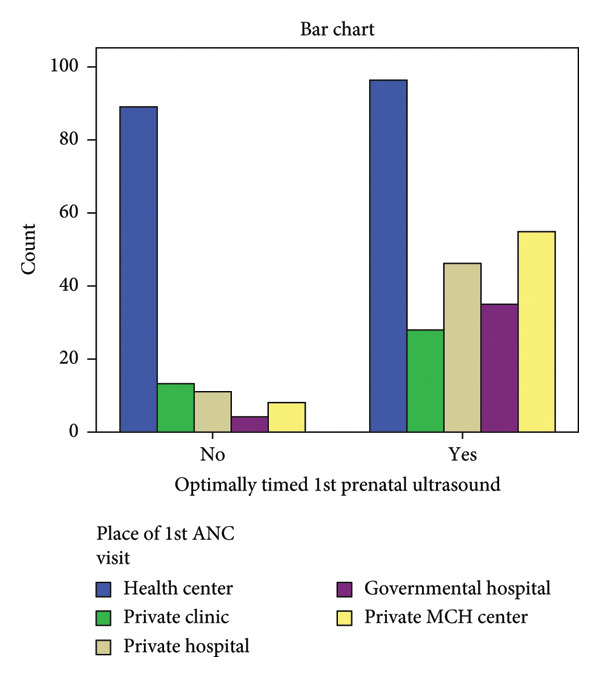
Proportion of optimally dated first ultrasound utilization according to place of 1^st^ prenatal contact.

From pregnant mothers who started their ANC at health centers, 48.1% did not have optimally timed 1^st^ prenatal US (Figure 1). 87.3% of those who started their ANC at private MCH centers and 89.7% of those who started their ANC at government hospitals had optimally timed 1^st^ prenatal US.

Multivariate regression analysis (Table 3) revealed that first place of ANC and the presence of prior major obstetric problem in prior pregnancies were associated with optimal timing of 1^st^ prenatal US. Compared to those having ANC at health centers, those who started their ANC at private clinic were 1.7 times (95 CI: 1.8–7.9) more likely to have optimally timed ANC. Those who had their 1^st^ ANC at private hospital were 3.2 times (95 CI: 1.8–7.9) more likely to have optimally timed 1^st^ prenatal care. Those who had their first ANC at private health institutions and government hospitals were much more likely to have optimally timed 1^st^ prenatal US with the following AOR for government hospital and private MCH centers: AOR = 7.4 (95 CI: 2.7–23) and AOR = 4.9 (95 CI: 2.8–14.1), respectively. Those who had previous major obstetric problem were 5.2 times (95 CI: 2.7–9.9) more likely to have optimally timed US than those without major previous obstetric problem.

**Table 3:** Factors associated with utilization of optimally dated first obstetric ultrasound among pregnant women who had their deliveries attended at three public hospitals in Ethiopia, 2020, *N* = 385.

Variable		No.	Percent	COR (95% CL)	AOR (95% CI)
1. Parity	Primigravid	155	40.3	0.56 (0.2–1.08)	
	Multigravid	230	59.7	1	

2. Known health problems before this pregnancy	Yes	187	48.5	0.98 (0.6–1.4)	
	No	198	51.5	1	

3. Health problems during this pregnancy	Yes	220	57.1	0.8 (0.5–1.3)	
	No	165	42.9	1	

4. Previous history of major obstetric problem during pregnancy	Yes	130	33.5	6.6 (3.5–12.2)	5.2 (2.7–9.9)
	No	100	26.5	1	1

5. Planned pregnancy	Yes	311	80.8	1.1 (0.6–1.9)	
	No	74	19.2	1	

6. Place of 1^st^ ANC visit	Health center	185	48.1	1	1
	Private clinic	41	10.6	1.99 (0.9–4)	1.7 (0.8–3.7)
	Private hospital	57	14.8	3.8 (1.8–7.9)	3.2 (1.5–6.8)
	Government hospital	39	10.1	8.1 (2.7–23.7)	7.4 (2.5–22.3)
	Private MCH center	63	16.4	6.3 (2.8–14)	4.9 (2.1–11.2)

## 4. Discussion

The primary objective this study was to determine the proportion of pregnant women who had optimal timing of first prenatal obstetric US and factors associated with it. We found that only two‐third of the study participants had optimally timed 1^st^ obstetric US. Place of first ANC contact (women who had their first ANC contact in private and government hospitals) and the presence of prior major obstetric problem were associated with optimally timed 1^st^ obstetric US.

WHO recommends every pregnant woman should have the 1^st^ obstetric US before 6^th^ month which corresponds to 24 weeks [18]. This criterion is clear and easy to implement as most of pregnant mothers in low‐middle income countries like Ethiopia count the duration of the pregnancy in months and most have difficulty remembering it in weeks. In our study, although most (67.5%) participants in our study had optimally timed 1^st^ US, significant number of mothers (32.5%) had no optimally timed 1^st^ prenatal US. This finding is similar to the findings of prior similar study conducted in Canada by Abdullah et al. [1]. However, there is difference between the two studies in the definition of optimally timed 1^st^ obstetric US: while Abdullah et al. defined optimally timed US as an US done between 11 and 22 weeks based on Society of Obstetricians and Gynaecologists of Canada (SOGC) recommendation, we defined optimally timed 1^st^ US based on WHO recommendation as an US done before 6^th^ month in our study. Although we could not compare our findings to the findings from other similar studies conducted in Africa, as there are no prior studies that looked into optimally timed first obstetric US as primary outcome, our main finding (one‐third of the participants did not utilize optimally timed first obstetric US) is alarming. Suprisingly, obstetric US services are available, free of charge, at public health institutions in Addis Ababa, where our study was conducted. We recommend proactive measures to be taken at different levels for improving early obstetric US utilization, including at patient level through creating awareness on the importance of having optimally timed first obstetric US.

Currently, there is strong evidence that supports the importance of US in enhancing fetomaternal health during pregnancy in low‐middle income countries [19, 20]. However, in most parts of the world, antenatal US is available only to a privileged few in urban centers, while the majority of the population living in rural areas have little or no access to diagnostic imaging services [21]. In Africa, a moderate utilization of obstetric US among pregnant mothers is on an increasing trend only after 2020 with patients’ good knowledge being among the factors associated with good utilization [22]. According to Ethiopia Demographic Health Survey (EDHS) 2019, 74% of women aged 15–49 with a live birth in the 5 years before the survey received ANC from a skilled provider for their most recent birth [23]. A study on utilization of prenatal US among pregnant women in public health institution in Ethiopia showed that 60.7% used prenatal US during their pregnancy [24]. Another similar study conducted in the capital Addis Ababa (Ethiopia) in public health institution showed that the utilization of prenatal US among pregnant women was 70.3% [25]. In the present study, of the factors which significantly affected the timing of optimally timed 1^st^ prenatal is the place of 1^st^ ANC: compared to those who started their ANC at health centers, those who started their ANC at private clinic were 1.7 times (95% CI: 1.8–7.9) more likely to have optimally timed ANC. Those who had their 1^st^ ANC at private hospital were 3.2 times (95% CI: 1.8–7.9) more likely to have optimally timed 1^st^ US. Similarly, those that had first ANC at government hospital (AOR = 7.4, 95% CI: 2.7–23) and private MCH centers (AOR = 4.9, 95% CI: 2.8–14.1) were more likely to utilize optimally dated first US.

Being among the first studies to report data on optimally dated first obstetric US from low‐income countries with proper sample size allocation and muticenter study design are the strengths of our study. Lack of random sampling technique and comparative analysis of perinatal outcomes between those who had optimally dated first obstetric US and those had are the main limitations of our study. The other limitation of this study is not considering the prevalence (*p*) in other international studies during sample size calculation, though there are no similar local or regional studies.

## 5. Conclusion

In this study, we found that one‐third of pregnant women did not utilize optimally timed first obstetric US despite obstetric US services being available at public health institutions. Place of first ANC contact and presence of prior major obstetric complication were associated with utilization of optimally dated first obstetric US. This findings demonstrates that availing US services alone even at no charge is not effective in ensuring universal utilization of early US by pregnant women unless and otherwise it is complemented with elevating patients’ awareness on the importance of having optimally dated obstetric US and improving practice of recommending early US by prenatal providers mainly midlevel health professionals, as they share a significant burden of providing ANC in public institutions in low‐middle income countries.

## Disclosure

All authors critically revised the article for intellectual content and gave final approval for submission for publication.

## Conflicts of Interest

The authors declare no conflicts of interest.

## Author Contributions

S.K. and A.A.A. were responsible for conception and development of the study project. S.K. and A.A.A. were responsible for data collection and data analysis. A.F.S., S.K., and E.M. were responsible for manuscript write‐up. The final manuscript was edited by A.F.S.

## Funding

No funding was received for this study.

## Data Availability

Data are available on request from the authors.
